# Polyploidization contributes to the forms, heights, and species richness of plants

**DOI:** 10.1093/hr/uhag223

**Published:** 2026-06-11

**Authors:** Jiangli Wang, Yao Wang, Chenxi Wang, Huilong Qi, Tianmeng Wang, Huizhe Zhang, Xiaochang Xu, Yuelong Jia, Fubo Hu, Bowen Song, Minran Yang, Song Ge, Tariq Shehzad, Andrew H Paterson, Xiyin Wang

**Affiliations:** School of Public Health, School of Life Science, College of Sciences, North China University of Science and Technology, Tangshan 063210, China; School of Public Health, School of Life Science, College of Sciences, North China University of Science and Technology, Tangshan 063210, China; School of Public Health, School of Life Science, College of Sciences, North China University of Science and Technology, Tangshan 063210, China; School of Public Health, School of Life Science, College of Sciences, North China University of Science and Technology, Tangshan 063210, China; School of Public Health, School of Life Science, College of Sciences, North China University of Science and Technology, Tangshan 063210, China; School of Public Health, School of Life Science, College of Sciences, North China University of Science and Technology, Tangshan 063210, China; School of Public Health, School of Life Science, College of Sciences, North China University of Science and Technology, Tangshan 063210, China; School of Public Health, School of Life Science, College of Sciences, North China University of Science and Technology, Tangshan 063210, China; School of Public Health, School of Life Science, College of Sciences, North China University of Science and Technology, Tangshan 063210, China; School of Public Health, School of Life Science, College of Sciences, North China University of Science and Technology, Tangshan 063210, China; School of Public Health, School of Life Science, College of Sciences, North China University of Science and Technology, Tangshan 063210, China; Institute of Botany, Chinese Academy of Science, Beijing 100093, China; College of Agronomy, Department of Crop Genetics and Breeding, Shandong Agricultural University, Taian 271018, China; Plant Genome Mapping Laboratory, University of Georgia, Athens, GA 30602, USA; School of Public Health, School of Life Science, College of Sciences, North China University of Science and Technology, Tangshan 063210, China

## Abstract

Whether plant life form influences the long-term persistence of polyploid lineages remains an important unresolved question in plant evolution. Previous studies have suggested that perennial herbaceous plants harbor higher proportions of neo-polyploids, yet comprehensive phylogenetic validation across paleopolyploid lineages using genomic data remains limited. The rapid accumulation of plant genome sequences, particularly from horticultural and agricultural species, provides an unprecedented opportunity to investigate the evolutionary relationships among polyploidization, plant growth form, and plant stature across seed plants. Here, we analyzed 351 seed plant genomes, including major cereals, vegetables, fruits, and their wild relatives, to investigate the long-term evolutionary associations between paleopolyploidization and plant phenotypic diversity. Phylogenetically informed comparative analyses showed that lineages experiencing more paleopolyploidization events were significantly more likely to exhibit herbaceous rather than woody growth forms. We further found that paleopolyploidization frequency was negatively associated with plant height across seed plants after accounting for phylogenetic relatedness. In addition, evolutionary rates, estimated using synonymous substitution rates (Ks), were associated with paleopolyploidization, plant growth form, and species richness. Herbaceous species exhibited higher Ks values than woody and vine species, and plant families with higher evolutionary rates tended to contain more species. Together, these findings provide a phylogenetically informed evidence that paleopolyploidization is closely associated with plant growth form, plant stature, and evolutionary rate, offering new insights into the long-term evolutionary consequences of genome duplication in seed plants.

## Introduction

Seed plants are the largest taxonomic group in the plant kingdom, distributed worldwide and occurring in woody, vine, and herbaceous forms [1]. They have two basic characteristics: (i) the presence of vascular tissues and (ii) the ability to reproduce through seeds. Angiosperms, or flowering plants, are the most diverse and widely distributed group of seed plants, with approximately 300 000 species. Monocotyledonous angiosperms (‘monocots’) are predominantly herbaceous, although some species are woody. Dicotyledonous angiosperms (‘dicots’ or often ‘eudicots’) can be woody, herbaceous, or vines. The remaining seed plants, gymnosperms, are mostly woody, with a few examples of vines, such as *Gnetum* species.

Polyploidy, or whole-genome duplication (WGD) [2–6], plays an important role in plant evolution [7]. Polyploids can be broadly divided into two categories: paleopolyploids and neopolyploids. Paleopolyploids are plants that have undergone WGD events in the distant past, typically millions of years ago. These events have been followed by extensive genetic and chromosomal changes, including gene loss, DNA fractionation, and the evolution of novel gene functions. As a result, the genomes of paleopolyploids often appear diploid-like in structure and behavior, despite their polyploid origin. Neopolyploids, in contrast, are plants that have experienced WGD events more recently, often within the last few hundred thousand years. These plants typically retain a high degree of genetic redundancy and chromosomal similarity to their diploid progenitors. Neopolyploids often exhibit immediate phenotypic changes and can provide insights into the early stages of polyploid evolution and adaptation [8].

Although classically viewed as an evolutionary dead end [9–12], it is now clear that all plant lineages have experienced polyploidization, and polyploidy contributed to the origin and diversification of many plant taxa [2, 13, 14]. Genome analysis of *Amborella* suggested that angiosperms shared a WGD at the phylum level, and most eudicots shared a hexaploid ancestor, whereas most monocots shared a tetraploid ancestor [15–19]. Phylogenetic studies and whole-genome sequencing of representatives from most large plant families (e.g. Poaceae, Fabaceae, Solanaceae) have shown that each family shares a specific WGD event [4, 20, 21]. Although recently formed polyploids have higher extinction rates than their diploid relatives [9], rare escapes from the ‘evolutionary dead end’ have made tremendous contributions to botanical diversity. Comparative surveys suggest that the frequency of recent (neo-)polyploidy differs among seed plants of different forms [22–24]. Compared to annuals, bulbous and woody plants, herbaceous perennial plants generally have the highest proportion of polyploids [23]. Several studies from different regions find perennials more likely to be polyploids, while annuals tend to be diploids. High polyploidy rates in eudicots are driven by herbaceous species [25], with no evidence of WGDs in woody dicots. The frequency of chromosome doubling in herbaceous dicots is more than six times higher than that of woody plants, accompanied by much higher chromosome instability [26]. In angiosperms from the Qinghai Tibet Plateau, a much higher proportion of polyploids were herbaceous plants (84.7%) than woody plants (12.6%) [27]. A similar finding was observed by comparing polyploid seed plants in the mountains of the Qinghai Tibet Plateau and the Pan-Arctic region [28].

Notably, the available genome sequences of seed plants are heavily enriched with horticultural and agronomic species that have been subject to long-term artificial selection and domestication [29]. These species, including major agronomic crops (e.g. *Triticum aestivum*, *Oryza sativa*, *Zea mays*) and economically important horticultural plants such as fruits or vegetables (e.g. *Citrullus lanatus*, *Cucumis sativus*, *Cucumis melo*), represent a unique evolutionary system in which natural evolutionary processes intersect with human-mediated selection [30]. Unlike purely wild systems, horticultural species offer well-documented evolutionary histories, extensive phenotypic records, and diverse life forms that have been shaped by both environmental adaptation and artificial selection for desirable traits [31]. Consequently, they provide an exceptional opportunity to investigate how paleopolyploidization and life form evolution interact under combined natural and artificial selective pressures [8].

An intriguing hypothesis is that plant longevity may influence the survival of genome duplication events. A low frequency of polyploidy in trees has been attributed to the impact of cell size on fiber formation. In bulb-forming plants, the low frequency of polyploidy has been attributed to the constraint imposed by large monoploid genome sizes [25]. Similarly, in annuals, strong selection against polyploid establishment may be due to the frequency-dependent success of homoploid mating and the importance of rapid development in colonizing species [32]. The rate of plant evolution is a pivotal topic in evolutionary biology, reflecting the velocity at which morphological, physiological, and genetic traits of species evolve over time. This rate is influenced by a complex interplay of environmental factors, such as climate and geography, and intrinsic biological factors, including polyploidization [33]. Synonymous substitution rates, which are putatively unaffected by natural selection, are commonly used to determine and date polyploidization or speciation events [34, 35]. Studies have shown significant differences in the rates of plant evolution, with some species evolving faster than others. For example, within the Poaceae family, *T. aestivum* (wheat), *Setaria italica* (foxtail millet), *Sorghum bicolor* (sorghum), *Z. mays* (corn), and *Brachypodium distachyon* have evolutionary rates 4.6% to 18.2% higher than *O. sativa* (rice), while the rate of *Hordeum vulgare* (barley) is even higher (28.1%–33.3%) [36]. In the Cucurbitaceae family, *C. lanatus* (watermelon) and *C. sativus* (cucumber) have 23.6%–27.4% higher evolutionary rates than *C. melo* (melon) [37]. Investigating these rates and their interplay with environmental factors may offer insight into mechanisms driving biodiversity and species’ adaptive potential. Together with other plants, these horticultural species have experienced accelerated evolutionary changes due to domestication bottlenecks and intensive selection, potentially amplifying rate differences observable across plant lineages [38]. However, comprehensive studies across a broad range of plant families are scarce, and a systematic approach to quantifying rate differences and environmental factors influencing them is yet to be established. All forms of plants frequently experience various abiotic stresses, such as drought, salinity, heat, and cold. To cope with these stresses and ensure normal growth, plants have evolved mechanisms to synthesize secondary metabolites, including phenols, terpenes, and alkaloids [39]. Secondary metabolic pathways in plants primarily encompass the phenylpropanoid, terpenoid, and alkaloid metabolic pathways, the former being particularly important [40] in the synthesis of lignin. Depending on the composition of three monomers (*p*-coumaryl alcohol, sinapyl alcohol, and coniferyl alcohol), lignin can be classified into *p*-hydroxyphenyl lignin (H lignin), guaiacyl lignin (G lignin), or syringyl lignin (S lignin) [41]. Lignin provides structural support to plant cell walls, enhances cell wall and stem strength, and plays a crucial role in plant growth and development [42].

Genome sequences now available for hundreds of seed plants enable broadening of surveys about the relationship between paleopolyploidization and life forms beyond neo-polyploids to paleopolyploids and diverse plant groups. Here, we utilized publicly available genome sequences—encompassing a substantial representation of horticultural species alongside their wild relatives—to further investigate this relationship, focusing on plant stature (height) as an integrative trait reflecting ecological strategy, resource allocation, and growth form. While plant height covaries with other life-history traits (e.g. perenniality, mating system) that we cannot fully disentangle, it provides a measurable and ecologically meaningful proxy for the relationship between plant architecture and genome duplication. Furthermore, we compare the evolutionary rates of 71 eudicots from 34 families, leveraging the shared core eudicot whole-genome triplication (ceWGT) as a common reference point. We establish a set of criteria that more accurately measure differences in species’ evolutionary rates. Although the available genomes still sample only a tiny fraction of seed plants, insight into patterns occurring over tens of millions of years contributes to understanding the interaction and evolution of plant forms and ecological changes.

## Results

### Dataset overview and phylogenetic framework

We compiled a dataset comprising 334 angiosperm species with well-assembled genome sequences (Table S1), representing 302 genera, 112 families, and 46 orders. The dataset included 254 eudicots (76.0%), 63 monocots (18.9%), and 17 basal angiosperms and magnoliids (5.1%) (Fig. 1). Phylogenetic relationships were reconstructed according to the Angiosperm Phylogeny Group IV (APG IV) classification system [43]. Among the sampled angiosperms, 147 species (44.01%) were woody and 187 species (55.99%) were herbaceous (Table 1). Vines were classified as woody or herbaceous according to stem texture. By reviewing published genomic studies, we determined the number of paleopolyploidization events experienced by each lineage since divergence from the angiosperm common ancestor.

**Figure 1:**
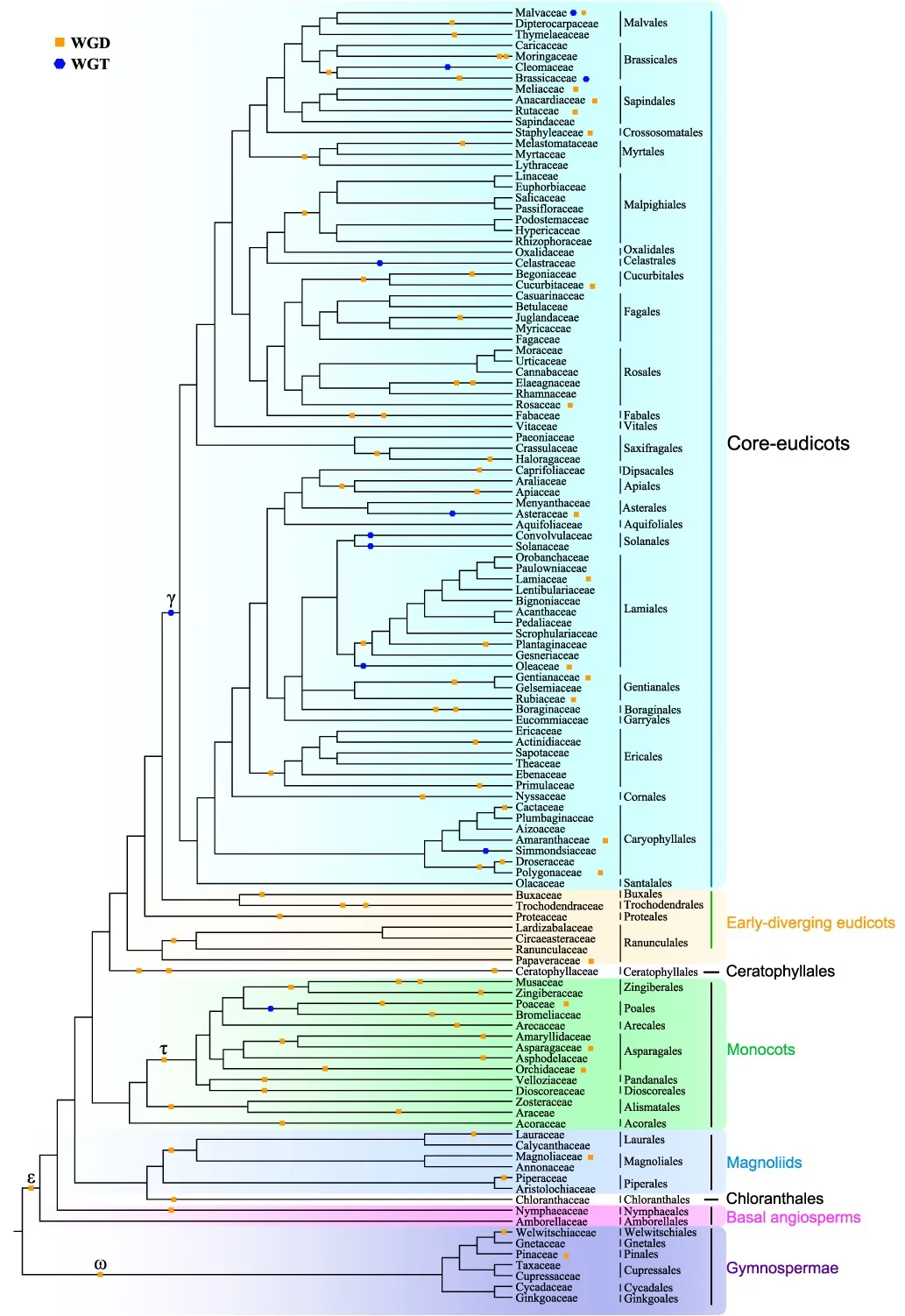
A phylogenetic tree showing paleo-polyploidization during the evolution of seed plants (based on APG IV). Squares represent WGD events, and hexagons represent WGT events. Gamma (γ) represents the ancient triplication event experienced by core eudicots; Tau (τ) represents the ancient duplication event shared by most monocots; Epsilon (ε) represents the ancient duplication event shared by angiosperms; Omega (ω) represents the ancient duplication event shared by gymnosperms.

**Table 1:** Chi-square test between the numbers of paleopolyploidization events and plant growth forms across seed plants and major angiosperm groups()

Plant group		The number of polyploidy events (%)						Total	χ^2^	*P*
		One	Two	Three	Four	Five	Six			
Seed plants	Woody plants	11 (84.62)	63 (73.26)	77 (44.77)	11 (18.97)	1 (5.00)	0 (0.00)	164 (46.72)	65.955	0.000**
	Herbaceous plants	2 (15.38)	23 (26.74)	95 (55.23)	46 (80.70)	20 (95.23)	2 (100.00)	187 (53.28)		
Subtotal		13	86	172	57	21	2	351		
Gymnosperms	Woody plants	10 (100.00)	7 (100.00)	–	–	–	–	17 (100.00)	–	–
	Herbaceous plants	0 (0.00)	0 (0.00)	–	–	–	–	0 (0.00)		
Subtotal		10	7	–	–	–	–	17		
Angiosperms	Woody plants	1 (33.33)	57 (72.15)	77 (44.77)	11 (18.97)	1 (5.00)	0 (0.00)	147 (44.01)	54.395	0.000**
	Herbaceous plants	2 (66.67)	22 (27.85)	95 (55.23)	46 (80.70)	20 (95.23)	2 (100.00)	187 (55.99)		
Subtotal		3	79	172	57	21	2	334		
Basal angiosperms	Woody plants	1 (33.33)	4 (57.14)	6 (85.71)	–	–	–	11 (64.71)	2.821	0.244
	Herbaceous plants	2 (66.67)	3 (42.86)	1 (14.29)	–	–	–	6 (35.29)		
Subtotal		3	7	7	–	–	–	17		
Monocots	Woody plants	–	0 (0.00)	5 (18.52)	0 (0.00)	1 (6.25)	0 (0.00)	6 (9.52)	4.839	0.304
	Herbaceous plants	–	2 (100.00)	22 (81.48)	17 (100.00)	15 (93.75)	1 (100.00)	57 (90.48)		
Subtotal		–	2	27	17	16	1	63		
Dicots	Woody plants	–	53 (75.71)	66 (47.83)	11 (26.83)	0 (0.00)	0 (0.00)	130 (51.18)	32.752	0.000**
	Herbaceous plants	–	17 (24.29)	72 (52.17)	29 (72.50)	5 (100.00)	1 (100.00)	124 (48.82)		
Subtotal		–	70	138	40	5	1	254		

Note: ** indicates P < 0.01 (highly significant); * indicates P < 0.05 (significant). "–" indicates not applicable.

To further examine broader evolutionary patterns, we also incorporated 17 gymnosperm species with publicly available high-quality genome assemblies, yielding a total dataset of 351 seed plant species belonging to 314 genera, 119 families, and 52 orders. The phylogenetic framework used in this study is shown in Fig. 1.

### Paleopolyploidization is associated with herbaceous growth form

To account for phylogenetic nonindependence among species, we first conducted phylogenetically informed comparative analyses examining the relationship between paleopolyploidization frequency and plant growth form across seed plants. Phylogenetic logistic regression revealed a significant negative association between woody growth form and the number of paleopolyploidization events (β = −0.756, SE = 0.159, *P* = 2.02 × 10^−6^), indicating that lineages experiencing more paleopolyploidization events were less likely to exhibit woody growth forms and were instead more frequently associated with herbaceous species (Fig. 2a).

**Figure 2:**
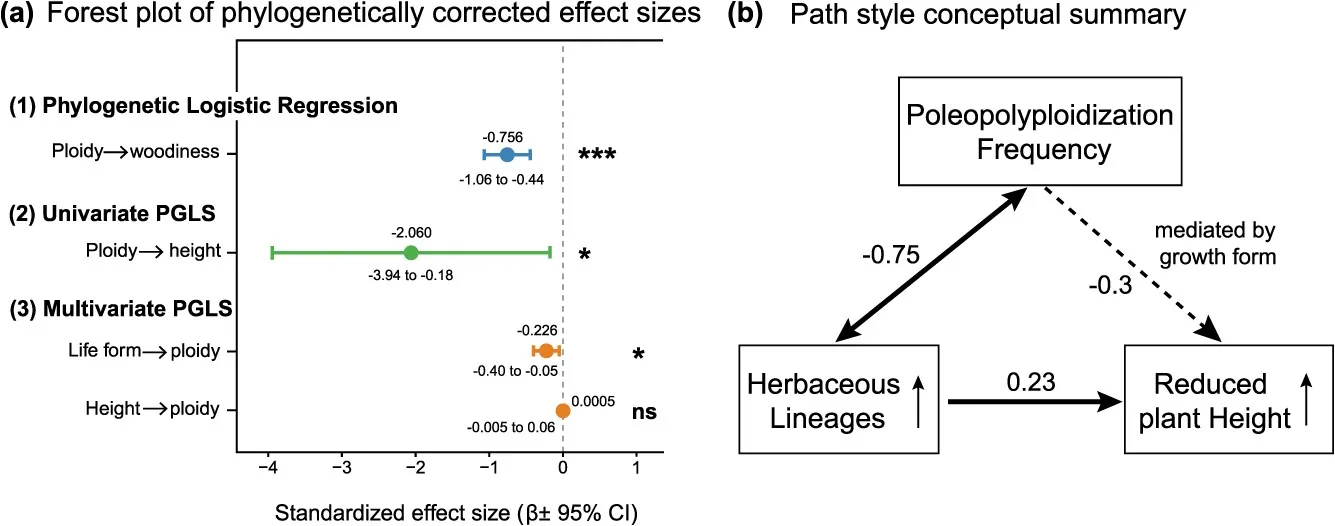
Phylogenetically informed associations among paleopolyploidization frequency, life form, and plant height. (a) Forest plot showing effect sizes (β ± 95% CI) from phylogenetic logistic regression, univariate PGLS, and multivariate PGLS models. Negative coefficients indicate that repeated paleopolyploidization events are associated with reduced woodiness and lower plant height. In the multivariate model, life form remained significant, whereas plant height was no longer significant. (b) Conceptual path-style summary illustrating that the negative association between plant height and paleopolyploidization frequency is largely mediated by the distinction between woody and herbaceous growth forms.

Conventional statistical analyses supported the same overall pattern. Across angiosperms, herbaceous species became progressively more prevalent with increasing numbers of paleopolyploidization events (Table 1). A chi-squared test strongly rejected the null hypothesis that paleopolyploidization frequency is independent of plant growth form (χ^2^ = 54.395, *df* = 5, *P* = 1.74 × 10^−10^). Similar trends were observed within eudicots, whereas monocots showed the same directional tendency despite weaker statistical support, likely reflecting more limited and unbalanced sampling. No significant relationship was detected among basal angiosperms and magnoliids.

Gymnosperms differed markedly from angiosperms in both paleopolyploidization frequency and life form composition. All sampled gymnosperms were woody species, precluding statistical evaluation of growth form effects within this group. When gymnosperms and angiosperms were analyzed together, encompassing 351 seed plant species, the relationship between paleopolyploidization frequency and plant growth form remained highly significant (χ^2^ = 65.955, *P* = 7.10 × 10^−13^; Table 1).

Overall, both phylogenetically informed and conventional analyses consistently supported an association between increased paleopolyploidization frequency and herbaceous growth forms.

### Paleopolyploidization frequency is negatively associated with plant height

Phylogenetic generalized least squares (PGLS) analysis demonstrated that plant height was significantly negatively associated with paleopolyploidization frequency across seed plants (β = −2.06, SE = 0.96, *P* = 0.033), indicating that lineages experiencing more paleopolyploidization events tended to be shorter after accounting for phylogenetic relatedness (Fig. 2a). The estimated Pagel's λ was 0.733 (95% CI, 0.490–0.876), supporting substantial phylogenetic signal in plant height variation.

To further disentangle the relationships among plant height, life form, and paleopolyploidization frequency, we fitted a multivariate PGLS model including both plant height and growth form. In this model, growth form remained a significant predictor of paleopolyploidization frequency (β = −0.226, SE = 0.089, *P* = 0.012), whereas plant height was no longer significant (*P* = .860), suggesting that the negative association between plant height and paleopolyploidization frequency is largely mediated by the distinction between woody and herbaceous growth forms (Fig. 2b).

Conventional correlation analyses recovered the same overall trend. Across 351 seed plant species, plant height showed a significant negative correlation with the number of paleopolyploidization events (correlation coefficient = −0.380, *P* = 1.63 × 10^−13^; Fig. 3, Table S2). Similar negative relationships were observed within woody plants, monocots, and eudicots, whereas herbaceous species showed a contrasting positive trend.

**Figure 3:**
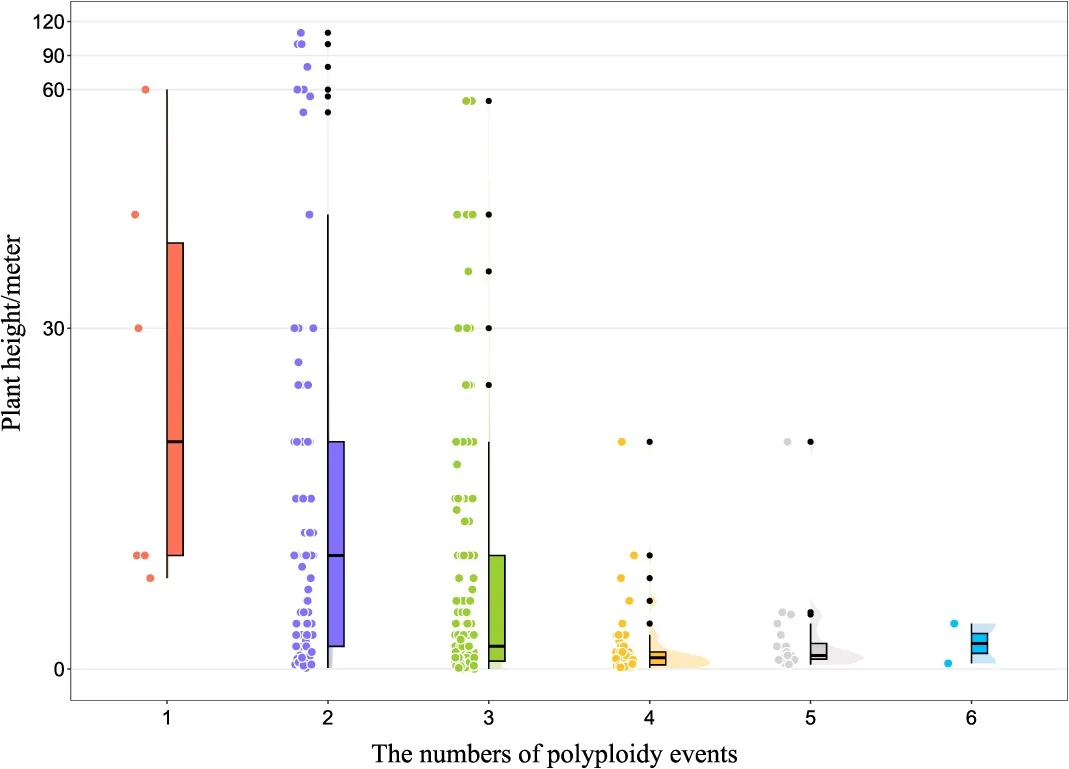
Relationship between plant height and paleopolyploidization frequency. The horizontal axis represents the number of paleopolyploidization events, and the vertical axis represents plant height.

Overall, both phylogenetically informed and conventional analyses consistently supported a negative association between plant height and paleopolyploidization frequency.

### Evolutionary rates are associated with paleopolyploidization, plant growth form, and species richness

To investigate variation in evolutionary rates among flowering plants, we analyzed 71 core eudicot species from 34 families that share the ancient ceWGT. Shared paleopolyploidization events provide a common temporal reference for comparing synonymous substitution rates (Ks) among species.

Phylogenetically informed analyses suggested a positive relationship between synonymous substitution rate and paleopolyploidization frequency. PGLS analysis showed a positive but nonsignificant association between Ks and the number of paleopolyploidization events (β = 0.058, SE = 0.036, *P* = 0.108), with an extremely high Pagel's λ estimate (0.998), indicating strong phylogenetic dependence in synonymous substitution rates. When both paleopolyploidization frequency and plant height were included as predictors in a multivariate PGLS model, paleopolyploidization frequency showed a marginally significant positive association with Ks (β = 0.066, SE = 0.036, *P* = 0.074), whereas plant height had no significant independent effect (*P* = .285) (Fig. 4a). Pagel's λ remained extremely high (0.997), further supporting strong phylogenetic structure in substitution rate variation.

**Figure 4:**
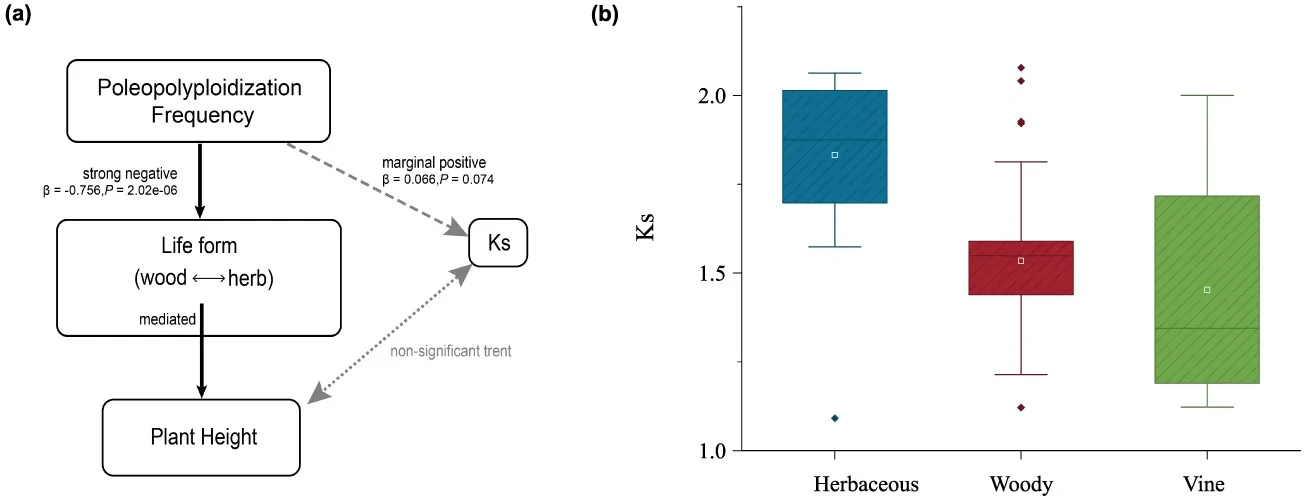
Integrated phylogenetic comparative network linking paleopolyploidization frequency, life form, plant height, and synonymous substitution rate (Ks). (a) The network summarizes the relationships among paleopolyploidization frequency, plant life form, plant height, and synonymous substitution rate (Ks) inferred from phylogenetically informed comparative analyses. Solid arrows indicate statistically supported associations, dashed arrows indicate marginal trends, and dotted relationships represent associations that became nonsignificant after multivariate correction. Repeated paleopolyploidization events are most strongly associated with herbaceous growth form, which largely explains the secondary association with reduced plant height. A weaker positive trend links repeated paleopolyploidization with elevated Ks. (b) Distribution of Ks values among herbaceous, woody, and vine plants. The boxplot illustrates the characteristics of the Ks values of species affected by different types of plants. The left boxplot represents the distribution of Ks values in herbaceous plants, the middle boxplot represents the distribution of Ks values in woody plants, and the right boxplot represents the distribution of Ks values in vine plants.

Conventional comparative analyses recovered similar trends. Among the 71 core eudicots, herbaceous species exhibited higher synonymous substitution rates (Ks = 1.836 ± 0.214) than woody species (1.534 ± 0.207) and vines (1.453 ± 0.386) (Fig. 4b). Herbaceous lineages accumulated 19.69% more synonymous substitutions than woody species and 26.36% more than vines. Herbaceous species also displayed lower variation in Ks values (coefficient of variation = 0.116) compared with woody plants (0.135) and vines (0.266).

Variation in evolutionary rates among plant families was closely associated with species richness. Using a phylogenetic framework of 34 core eudicot families (Fig. S1), we calculated the average synonymous substitution rate (Ks) for representative species within each family and performed regression analyses against family-level species richness. These analyses revealed a strong positive relationship between average family Ks values and species number (*R^2^* = 0.8993; Fig. 5a).

**Figure 5:**
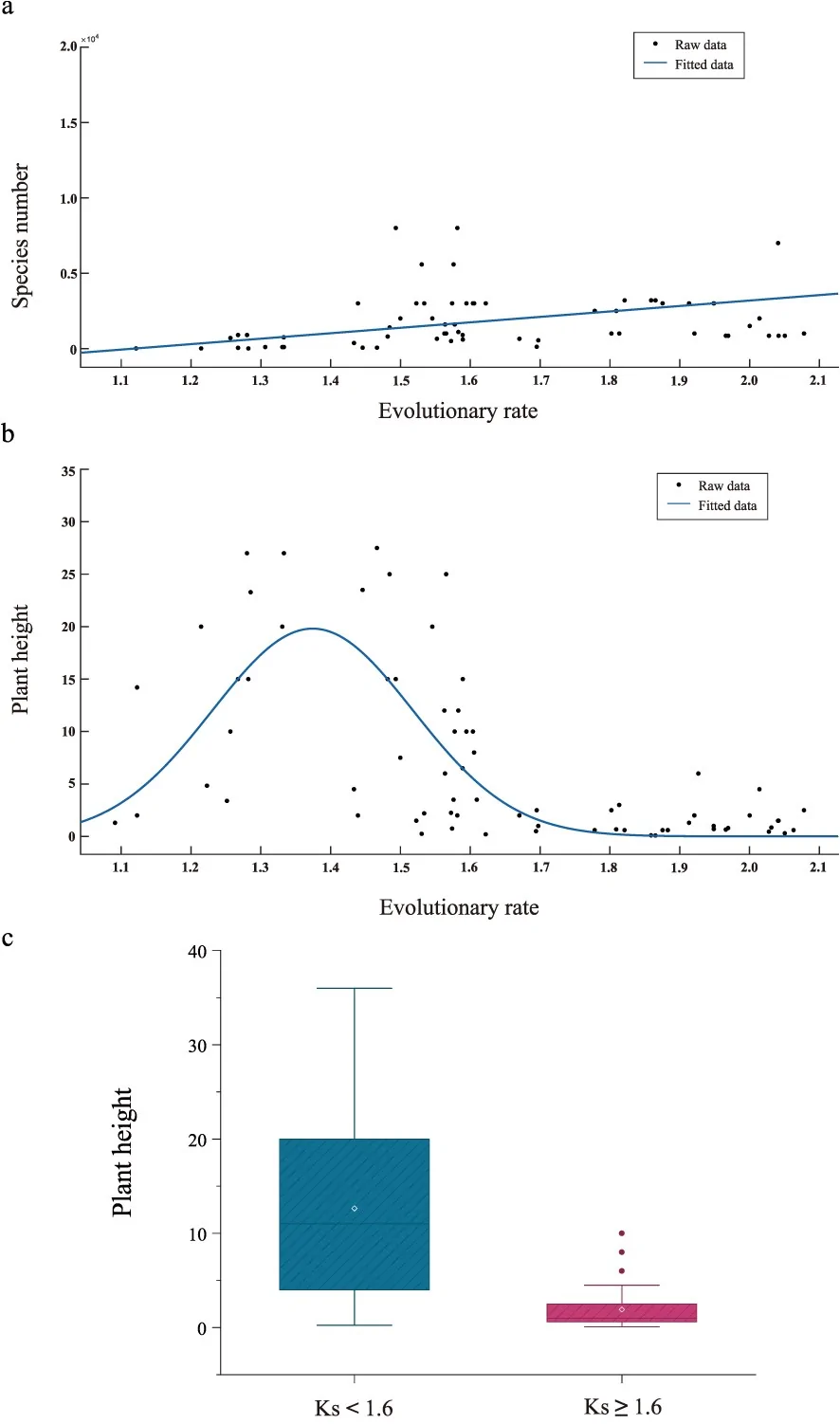
Relationships among evolutionary rate, species richness, and plant height. (a) Regression analysis between species richness and evolutionary rate. (b) Curve fitting of plant height and evolutionary rate. Dots represent the original data, and the blue line represents the fitted curve. (c) The impact of evolutionary rate on the distribution of plant height. The left box plot represents the distribution of plant heights in the group with slower evolutionary rates (Ks < 1.6); the right box plot represents the distribution of plant heights in the group with faster evolutionary rates (Ks ≥ 1.6).

Evolutionary rate was also associated with plant stature (Fig. 5b and c). Although the relationship followed an approximately normal distribution pattern (*R^2^* = 0.9326), a distinct shift in plant height was observed around Ks = 1.6. Species with Ks values below 1.6 had an average height of 12.652 m, whereas species with Ks values equal to or greater than 1.6 averaged only 1.913 m, representing a significant difference between the two groups (*t*-test, *P* < 0.05).

Overall, phylogenetically informed and conventional analyses consistently suggested that elevated evolutionary rates are more frequently associated with herbaceous and paleopolyploid-rich lineages.

## Discussion

Seed plants and especially angiosperms have been strongly affected by recursive polyploidizations [2, 3, 6], each causing extensive genome instability including large-scale chromosomal rearrangement [44–46], removal or translocation of (duplicated) genes [11, 47], illegitimate recombination [48–50], DNA segmental deletion, and mutation and other changes. Survival of these changes would require rewiring of regulatory pathways and networks [51]. Most of these events exhibit independent functional effects [5, 52]. While the overwhelming majority of polyploidies cannot survive the competition with their diploid progenitors, even subtle ecological advantages (or smaller disadvantages) may profoundly affect the abundance of polyploids in different taxa.

Here, by considering both paleopolyploids and neopolyploids, we found a significant correlation between incidences of polyploidization and life forms across seed plants and most subgroups. Large-statured plants are less likely affected by additional polyploidization events than short-statured plants. This suggests that recursive genome doubling is associated with specific life forms in herbaceous plants, though the underlying mechanisms—whether through competitive advantage against diploid progenitors, enhanced stress tolerance, or other ecological factors—remain to be fully elucidated. We found support for a specific environmental association: cold stress, evidenced by the positive correlation between CRG protein content and polyploidy frequency (correlation coefficient = 0.355; Table S4). However, we emphasize that our analysis did not test whether polyploid lineages show broader geographical distributions in harsh environments, higher species diversification rates within families, or increased heritable phenotypic variance—key predictions of the “polyploid advantage” hypothesis that require direct empirical validation. The association between polyploidy and cold tolerance, while statistically robust, should be interpreted as correlational rather than causally demonstrative of adaptive superiority in extreme environments.

Our survey of 351 seed plant species is enriched with horticultural crops and their wild relatives (197 wild species, 56.1%; Table S1), reflecting the current state of plant genomics where economically important cultivated species and their wild relatives dominate available high-quality genome assemblies. Such a dataset presents both unique opportunities and interpretive challenges. On one hand, horticultural species provide well-documented evolutionary histories and extensive phenotypic records, enabling robust correlations between genome evolution and life form traits [53]. On the other hand, these species have experienced strong artificial selection and domestication bottlenecks, which may amplify or alter patterns observed in natural systems [38]. For instance, the faster evolutionary rates and recurrent polyploidization in herbaceous eudicots—prominently represented by crops in Solanaceae, Fabaceae, and Brassicaceae—may partly reflect human-mediated selection for rapid growth, high yield, and stress tolerance, rather than purely natural adaptive processes [8, 54]. Consequently, the correlations we observe between polyploidization, short stature, and accelerated molecular evolution likely represent the combined effects of natural selection and artificial breeding. Disentangling these contributions requires future comparative analyses between horticultural lineages and their wild progenitors with equivalent ploidy histories.

These herbaceous perennials have evolved various strategies to cope with environmental stresses, including cold, for example, through the development of underground storage organs such as tubers and rhizomes. Recent large-scale biogeographic analyses further support a close association between polyploidy and cold environments [55]. Polyploid plants are disproportionately more frequent in colder climates and at higher latitudes, particularly in tundra, boreal, and montane ecosystems. Importantly, the effect of climate on global polyploid distribution appears to be mediated largely through plant life-form composition, especially the increased abundance of perennial herbaceous species in cold environments. This interpretation is highly consistent with our observation that lineages experiencing more paleopolyploidization events are more frequently associated with herbaceous growth forms and reduced plant stature.

Cold stress has also been associated with the occurrence of polyploidization based on both geological surveys and genomic analysis [56–59]. Harsh and fluctuating environments may increase the formation frequency of unreduced gametes, thereby facilitating the establishment of new polyploid lineages [55]. Consistent with this hypothesis, we found a positive correlation (correlation coefficient = 0.355) between the content of CRG protein sequences and the number of polyploid events occurring in plants (Table S4). Doubled genomes may enhance the robustness of the regulatory networks to withstand harsh environmental changes [60]. In contrast, most woody plants are perennial and typically occupy relatively stable environments, likely reflecting natural selection favoring long-lived individuals with strong physical protection and developmental stability.

Physically sturdy plants with strong stumps and thick protective bark, such as trees and vines, often have much simpler genomes than herbaceous plants. Woody eudicots, such as *Vitis vinifera* (grape), *Pyrus betulifolia* (pear), *Prunus persica* (peach), and *Coffea canephora* (coffee), shared a common hexaploid ancestor 130 million years ago, but have not experienced further polyploidization, in contrast to many eudicot relatives with more complicated genomes in Solanaceae, Fabaceae, or Brassicaceae families [20, 21, 61]. Gymnosperms and many woody dicots have secondary growth that provides physical barriers against various environmental stresses [62–64], possibly accompanied by prolonged meiosis, multiple pairings of homeologous chromosomes, and changes in reproductive forms (Fig. 6). In contrast, most monocot plants have no secondary growth, and few are woody, perhaps conferring a stronger selective advantage to the genetic and regulatory complexity resulting from further polyploidization (Fig. 6).

**Figure 6:**
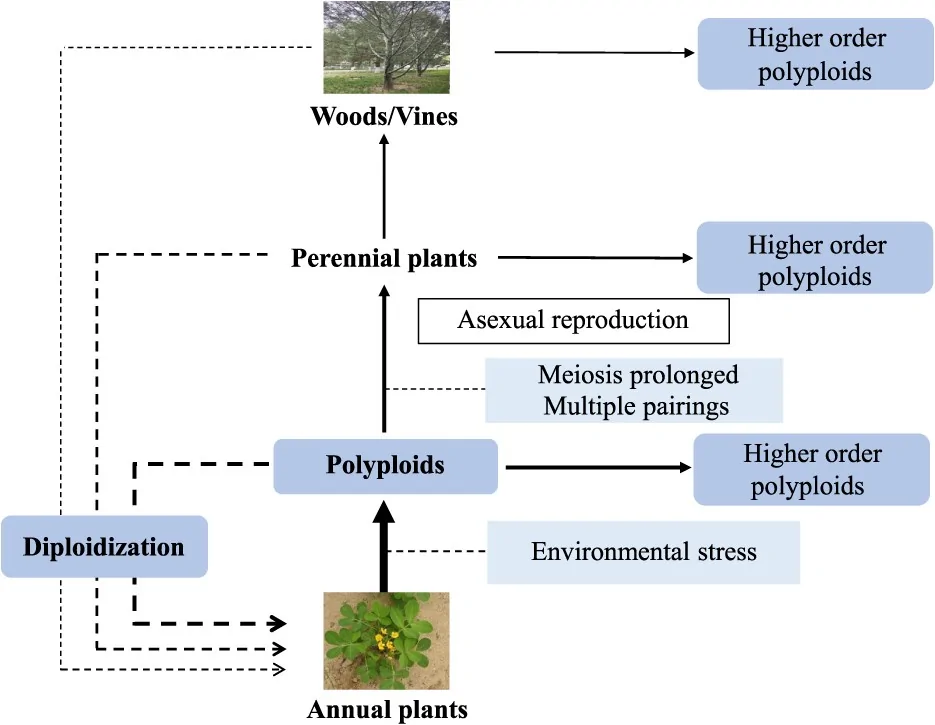
Polyploidization and plant form evolution. The thickness of arrowed lines shows the likelihood of related changes.

A strong positive correlation between stature and polyploidization made herbaceous plants a striking outlier to the negative relationship found for woody plants, monocots and eudicots (Table S2). One contributing factor may be that herbaceous neo-polyploids, especially allopolyploids, compared to their diploid relatives, could have larger biomass and therefore grow stronger and higher due to intergenomic heterosis. In addition, autopolyploids have also been reported to exhibit larger biomass than their diploid counterparts [65, 66]. Because of more extensive divergence between the respective subgenomes, allopolyploids have higher hybrid vigor or heterosis than autopolyploids, though the two forms of polyploidy are inferred to occur at similar frequency in natural populations [5, 67]. Autopolyploids may also acquire some hybrid vigor after recovering diploid inheritance, as highly similar subgenomes could experience unbalanced DNA mutations and/or complement gene losses, eventually resulting in diverged subgenomes. Therefore, owing to hybrid vigor, a positive correlation between stature and polyploidization is observed with herbaceous plants, often being neo-polyploids or polyploids recently formed. Comparatively, woody plants are often rather ancient polyploidies, perhaps lacking the hybrid vigor of neo-polyploids. A negative correlation between stature and polyploidization in a broader survey of plant groups, which includes not only herbaceous plants but also woody plants, should have resulted from biomass accumulation over the years that lengthens the stature.

Polyploidization multiplies thousands of genes simultaneously, and these genes are often subject to fast genetic changes, e.g. extensive gene losses, due to postpolyploidy genome instability likely caused by homologous chromosome recombination, creating potential for genetic innovation through duplication–degeneration–complementation processes [7, 68, 69]. Our study indicates that (additional) polyploidization events can accelerate the evolutionary rate of genes, as measured by synonymous substitution rates (Ks). This elevated molecular evolutionary rate may reflect relaxed purifying selection on duplicated gene copies, rather than necessarily indicating enhanced adaptive potential or increased heritable variance for quantitative traits. Since the considered polyploidization events are (tens and even hundreds of) millions of years old, this demonstrates that the occurrence of polyploidization has left lasting signatures in plant genomes, though the translation of these molecular patterns into ecological success or morphological diversification requires further investigation integrating population genetics and ecological data. Woody plants are often omitted from neo-polyploidization studies. However, as a complementary avenue to maintain appreciable levels of genetic diversity, the accumulation of transposons and other DNA repeats occurs, enlarging their genome sizes. As previously reported, compared with herbaceous plants, woody plants generally exhibit higher genetic diversity within species (populations) and significantly lower genetic differentiation among populations [70]. This intrinsic genetic diversity in woody plants can be partly attributed to the activities of transposons and ectopic DNA recombination between neighboring repetitive or genic elements, which generate abundant intraspecific variation over long life spans [71]. However, the magnitude of these changes is still not comparable to that occurring in neo-polyploids, in which thousands of duplicated genes can be rapidly lost, silenced, or fractionated in a nearly stochastic manner over short evolutionary timescales [72]. Therefore, woody plants often have relatively few close relatives within their genera. It is noteworthy that this pattern may be attenuated in horticultural woody species (e.g. Vitis, Pyrus, and Prunus), where artificial selection and clonal propagation have preserved substantial standing variation and delayed lineage turnover, contrasting with wild woody perennials in which low diversification rates and long generation times predominate [73, 74].

Several limitations of this study should be acknowledged. First, our dataset is biased toward species with available high-quality genomes, and we used species-average height values without accounting for intraspecific cytotype variation; both factors may skew patterns relative to natural plant communities. Future studies incorporating population-level cytotype data would complement our broad-scale approach. Second, our analyses establish correlations between polyploidy, life form, and molecular evolutionary rates, but do not demonstrate causation or test predictions about adaptive radiation, geographical range expansion, or heritable phenotypic variance central to the hypothesis that polyploidy promotes diversification. Future research should explicitly compare horticultural and wild lineages with matched ploidy histories and integrate genomic analyses with ecological distribution modeling and quantitative genetic experiments to assess whether the molecular patterns we observe translate into measurable fitness advantages and enhanced evolvability. Despite these limitations, our survey across 351 species from 34 families provides a phylogenetically informed framework for understanding the evolutionary associations among paleopolyploidization, plant growth form, plant stature, and evolutionary rate, offering new insights into the long-term consequences of genome duplication in seed plants.

## Conclusion

In this study, we analyzed 351 seed plant genomes to investigate long-term evolutionary associations among paleopolyploidization, plant growth form, plant stature, and evolutionary rate. Phylogenetically informed analyses consistently showed that increased paleopolyploidization frequency is associated with herbaceous growth forms and lower plant height across seed plants. We further found that evolutionary rates are associated with paleopolyploidization, plant growth form, and species richness, highlighting the importance of genome duplication in shaping plant evolutionary diversity. These findings provide a phylogenetically informed framework for understanding the long-term evolutionary consequences of paleopolyploidization and offer new insights into the relationships between genome evolution and plant phenotypic diversification.

## Materials and methods

### Data sources

Based on the species list of sequenced plants included in the plant genome website Plabipd (https://www.plabipd.de/plant_genomes_pa.ep), seed plants with high-quality reference genomes were obtained. The data mainly include genome annotation files (GFF), translated protein files (PEP), and coding sequence files (CDS). Python scripts were used to process these data into a unified format.

The final dataset comprised 351 seed plant species from 34 families, reflecting the current bias in plant genomics toward horticultural and economically important species. Specifically, 197 species (56.13%) are categorized as wild species, while 294 species (83.76%) are classified as horticultural crops (the remaining 57 species, 16.24%, are nonhorticultural taxa), accompanied by supporting rationales for horticultural crop designation in the third column of the table.

Relevant life history and plant height information for each species were obtained from the Plant Science Data Center (https://www.plantplus.cn/en). The average height of a plant was used in the statistical test. For species with documented intraspecific ploidy variation, height values represent the predominant or reference-genome cytotype, as our interspecific comparative framework focuses on paleopolyploidy events shared at family or ordinal level rather than intraspecific neo-polyploidy. We acknowledge that height may vary among cytotypes within mixed-ploidy species, but such variation operates at a microevolutionary scale distinct from the deep-time patterns we investigate. Information on paleopolyploidization events was compiled from previously published genomic and phylogenomic studies. These studies identified WGD or whole-genome triplication (WGT) events based on multiple lines of evidence, including synonymous substitution (Ks) distributions, syntenic block analyses, and phylogenomic inference. For each species, the number of ancient polyploidization events since divergence from the angiosperm common ancestor was determined by integrating published WGD reconstructions with lineage-specific genome analyses. Major shared events such as the ε duplication in angiosperms, τ duplication in monocots, and the γ hexaploidization in core eudicots were treated as common reference points.

### Tree-building methods

The phylogenetic trees presented in Figs 1 and S1 were constructed using the protein sequences of involved species. Relevant dataset includes 351 seed plant species with well-assembled genome sequences, covering 314 genera, 119 families, and 52 orders. The classification system used is the APG IV [43]. The phylogenetic trees were constructed using OrthoFinder [75], which is a powerful tool for inferring orthogroups and constructing phylogenetic trees from whole-genome sequences. The resulting trees were visualized using MEGA [76].

### Phylogenetic comparative analyses

To account for phylogenetic nonindependence among species, we incorporated phylogenetically informed comparative analyses throughout all trait association tests. Because branch lengths were unavailable in the original species tree, branch lengths were assigned using Grafen’s method implemented in the compute.brlen function in the R package ape under R version 4.5.3.

The relationship between plant life form (woody vs. herbaceous) and the number of paleopolyploidization events was first evaluated using phylogenetic logistic regression implemented in the phyloglm function of the phylolm package in R v4.5.3, using the bias-reduced logistic_IG10 method.

To assess continuous trait associations, we conducted PGLS analyses using the caper package (v1.0.3) in R v4.3.2, with phylogenetic covariance modeled by maximum-likelihood estimation of Pagel's λ. Specifically, we tested the relationships between paleopolyploidization frequency and plant height, as well as between paleopolyploidization frequency and synonymous substitution rate (Ks). For the Ks analyses, only species with available Ks estimates were retained in a filtered dataset, and the phylogeny was pruned to include the corresponding taxa.

To further evaluate trait interdependence, we additionally fitted multivariate PGLS models. These included (i) a model testing whether plant height and life form jointly predicted paleopolyploidization frequency, and (ii) a model assessing whether the association between Ks and paleopolyploidization frequency remained after accounting for plant height. Together, these phylogenetically corrected univariate and multivariate analyses allowed us to disentangle the interdependent relationships among plant life form, height, evolutionary rate, and repeated paleopolyploidization events.

### Statistical tests

To test whether paleopolyploidy events were nonrandomly distributed across plant life forms, we performed chi-squared (χ^2^) tests of independence using SPSS Statistics (version 25.0, IBM). We tested associations between life form (woody vs. herbaceous) and WGD event frequency in six lineages (seed plants, gymnosperms, angiosperms, basal angiosperms, monocots, and eudicots) using 2 × *k* contingency tables via the Crosstabs procedure. Table dimensions varied by lineage (*k* = 2–6) reflecting differential WGD event spectra. Pearson's χ^2^ test was used when ≥80% of expected cell frequencies were ≥5; otherwise, we applied Monte Carlo exact tests or category merging. Gymnosperms lacked herbaceous species (structural zero) and were analyzed separately. Bonferroni correction was applied across six tests (α_adj = 0.008). Effect sizes were quantified by Cramer's V.

### Inference of evolutionary rates

We adopted the colinearity inference toolkit WGDI [77] to identify colinear gene pairs within genomes. Subsequently, we estimated the synonymous nucleotide substitutions (Ks) between colinear duplicate genes. Considering that the plants in our study have experienced at least one polyploidization event beyond the ceWGT, we utilized homologous gene dot plots to discern the ceWGT-derived duplicates from those produced by additional polyploidization events. This approach is crucial for accurately differentiating paralogous genes originating from ceWGT and the other polyploidization events (Fig. S2). For instance, beyond the shared ceWGT initially identified in grape, mango has undergone an additional WGD event. Due to the frequent presence of long tails in Ks distribution curves, we extracted the median Ks value from each identified colinear gene block, which often offers a statistically robust inference. We characterized the distributions of these Ks medians across all colinear blocks from each genome. The resulting Ks distributions were then modeled based on the principle of normal distribution to determine the peak of the Ks value (Table S3).

### Identification of CRGs

From the Arabidopsis Genome Website (TAIR) (http://www.arabidopsis.org/), we downloaded 115 protein sequences of cold resistance genes (CRGs), which were used to search in each genome to find putative homologous genes (BLASTP program, with *E*-value set to 1e−5).

## Supplementary Material

Web_Material_uhag223

## Data Availability

No new data were generated or analyzed in support of this research.
