# Supplementary material for: Polyploidization contributes to the forms, heights, and species richness of plants
Source: Hortic Res. 2026 Jun 11;13(11):uhag223. doi: 10.1093/hr/uhag223 (PMC13722872; doi:10.1093/hr/uhag223)
Supplement: Supplementary file 1 [file web_material_uhag223.zip › Supplementary Figures.docx]

**Polyploidization contributes to the forms, heights, and species richness of plants**

**Supplemental Figure S1-S2**

**Figure S1. Phylogenetic tree of estimated species numbers within a family.** The tree includes 34 families from 16 plant orders and the number of species contained within each family. The green lightning bolt symbol (γ) represents the whole-genome triplication (ceWGT) that the core eudicot ancestors experienced together.

**Figure S2. Examples of Ks distribution.** (a), distribution of Ks among collinear genes from *Vitis vinifera*; (b), distribution of Ks among collinear genes from *Mangifera indica*. (c), distribution of Ks among collinear genes from *Actinidia chinensis*. (d), collinear gene blocks of the *V. vinifera* genome; (e), collinear gene blocks of the *M. indica* genome; (f), collinear gene blocks of the *A. chinensis* genome;
